# DeWitt County Medical Society

**Published:** 1866-05

**Authors:** C. Goodbrake

**Affiliations:** Secretary


### PROCEEDINGS OF THE DeWITT COUNTY MEDICAL SOCIETY.

The Society met in Annual Session, in Clinton, at the office of Dr. Shurtleff, on Monday, the 2d day of April, 1866, Dr. Tyler in the Chair.

The minutes of the previous meeting were read and approved.

On motion of Dr. Madden, Dr. Drombolone and Dr. Jno. A. Edmiston were invited to participate in the proceedings of the meeting.

The election of officers being in order, the following gentlemen were elected for the ensuing year:—

*President*—Dr. John Wright.

*Vice-President*—Dr. B. K. Shurtleff.

*Secretary*—Dr. C. Goodbrake.

*Censors*—Dr. J. H. Tyler, Dr. R. T. Richards, and Dr. T. W. Davis.

*Delegates to the Illinois State Medical Society*—Dr. R. T. Richards, Dr. B. K. Shurtleff, and Dr. T. W. Davis.

*Delegates to the American Medical Association*—Dr. B. S. Lewis and Dr. T. K. Edmiston.

Dr. Tyler, the retiring President, then delivered an address on the diseases most prevalent in DeWitt County during the past year, for which he received the thanks of the Society.

Dr. John A. Edmiston, of Clinton, and Dr. Charles W. Drombolone, of Marion, having been proposed for membership, and the Censors having reported favorably, they were unanimously elected members of the Society.

Dr. Z. H. Madden reported a case of intermittent fever, followed by partial paralysis, which called up a discussion, in which most of the members present participated.

The essayists appointed at the last meeting, having failed to fulfil their duty, were continued, and the President appointed Drs. J. A. Edmiston and C. W. Drombolone to read each an essay at the next meeting.

Cholera was chosen as the subject for discussion at the next meeting.

On motion, it was

*Resolved*, That in view of the probable visitation of cholera, this Society earnestly recommend that the Board of Trustees of the Town of Clinton take the necessary steps to remove all filth and garbage from the public square, streets, and alleys of said town, and that it pass an ordinance compelling the citizens to remove from their premises all offal, manure, and other kinds of filth; and, so far as possible, to whitewash all unpainted fences and outhouses; also to apply freely of lime to sinks, sewers, and privies; and that the carrying out of the same measures be recommended to the citizens of the several unincorporated towns in the County.

On motion, the Secretary was ordered to furnish copies of these proceedings to the *Chicago Medical Examiner*, *Clinton Public*, and *Chicago Medical Journal* for publication.

On motion, the Society adjourned, to meet in Quarterly Session at Clinton, on the first Monday of July next, at 10 o'clock A.M., at Dr. Shurtleff's office.

C. GOODBRAKE, *Secretary.*

